# Study on the Interaction Mechanism of Theaflavin with Whey Protein: Multi-Spectroscopy Analysis and Molecular Docking

**DOI:** 10.3390/foods12081637

**Published:** 2023-04-13

**Authors:** Jia Xu, Yi Huang, Yang Wei, Xinchu Weng, Xinlin Wei

**Affiliations:** 1 School of Environmental and Chemical Engineering, Shanghai University, Shanghai 200444, China; 2 School of Agriculture and Biology, Shanghai Jiao Tong University, Shanghai 200240, China

**Keywords:** theaflavin, whey protein, interaction, spectroscopy analysis, molecular docking

## Abstract

The interaction mechanism of whey proteins with theaflavin (TF1) in black tea was analyzed using multi-spectroscopy analysis and molecular docking simulations. The influence of TF1 on the structure of bovine serum albumin (BSA), β-lactoglobulin (β-Lg), and α-lactoalbumin (α-La) was examined in this work using the interaction of TF1 with these proteins. Fluorescence and ultraviolet-visible (UV-vis) absorption spectroscopy revealed that TF1 could interact with BSA, β-Lg and α-La through a static quenching mechanism. Furthermore, circular dichroism (CD) experiments revealed that TF1 altered the secondary structure of BSA, β-Lg and α-La. Molecular docking demonstrated that the interaction of TF1 with BSA/β-Lg/α-La was dominated by hydrogen bonding and hydrophobic interaction. The binding energies were −10.1 kcal mol^−1^, −8.4 kcal mol^−1^ and −10.4 kcal mol^−1^, respectively. The results provide a theoretical basis for investigating the mechanism of interaction between tea pigments and protein. Moreover, the findings offered technical support for the future development of functional foods that combine tea active ingredients with milk protein. Future research will focus on the effects of food processing methods and different food systems on the interaction between TF1 and whey protein, as well as the physicochemical stability, functional characteristics, and bioavailability of the complexes in vitro or in vivo.

## 1. Introduction

Tea is one of the most popular beverages in the world and contains a variety of active ingredients. Tea has a long history, but it also has medical and health benefits [1]. Black tea is one of the most widely consumed commercial tea, accounting for approximately 78% of the total consumption of the tea beverage industry [2]. Theaflavins (TFs), the primary polyphenols in black tea, have attracted a lot of interest due to their distinct physiological and functional properties. The level of TFs in black tea is about 0.5–3%, which plays a vital role in the quality and determines the black tea’s fresh and mellow taste [3]. In recent years, studies revealed that TFs have a variety of pharmacological functions and healthcare effects, such as lowering blood pressure, lowering blood sugar, reducing fat, anti-oxidation, anti-tumor and protecting bone health [4,5,6,7,8,9,10]. These findings suggested that TFs could be used as nutritional supplements in functional foods.

**Figure 1:**
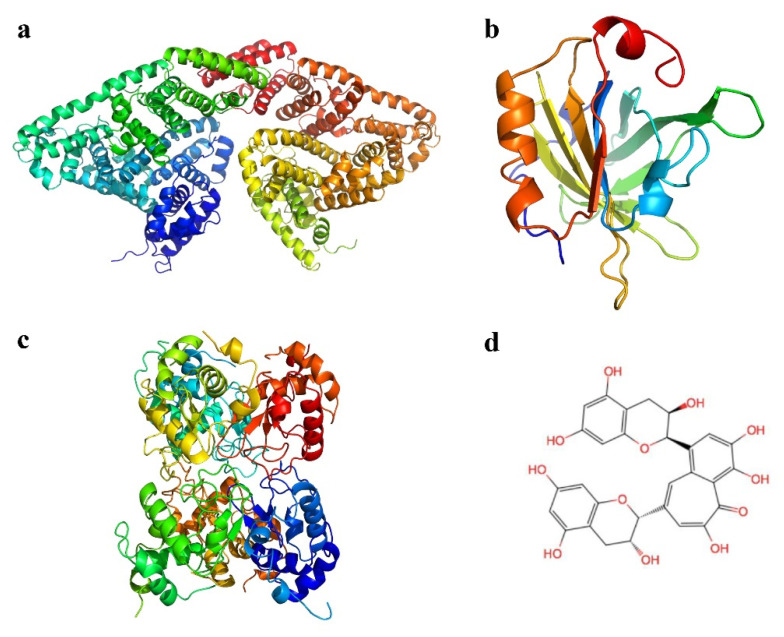
Tertiary structure of BSA (**a**), β-Lg (**b**), α-La (**c**) and chemical structure of theaflavin (**d**).

TFs are a class of chemicals with a benzodiazepine structure that are primarily formed through the oxidative condensation of catechins and their derivatives, which is catalyzed by enzymes including polyphenol oxidase [11]. Theaflavin (TF1), theaflavin-3-gallate (TF2), theaflavin-3′-gallate (TF3), and theaflavin-3, 3′-digallate (TFDG) are the most important TFs in black tea [12]. The quantity and positioning of the gallic acyl groups in the precursor catechins determine the structure of the theaflavins. Therefore, its physicochemical properties are similar to catechins, which are active, easily oxidised and unstable [13]. The most popular ligand in previous studies, TF1, was employed in this work, and its chemical structure was depicted in Figure 1d [12]. Several factors, including structural instability, efflux transporters, and cell metabolism, have been identified as influencing TF bioavailability [14]. Hence, TFs’ relatively unstable nature and low bioavailability are two important disadvantages that may limit their application.

Bovine whey protein accounts for approximately 20% of total milk protein, it is a recycled by-product of the cheese production process and is nutritionally rich but relatively affordable [15]. The bovine whey protein is compact globular proteins isolated from milk using advanced extraction methods, mainly including β-lactoglobulin (β-Lg), α-lactoalbumin (α-La), lactoferrin, bovine serum albumin (BSA), immunoglobulin and growth factor. Whey protein’s distinct amino acid sequence and three-dimensional structure confer diverse functional properties [16]. Due to its high bioavailability, whey protein is widely utilized in the food industries as an emulsifier for encapsulating active functional ingredients [17,18,19]. BSA has a molecular mass of 66.3 kDa and accounts for around 5% of whey protein. It can bind a wide range of intrinsic and extrinsic substances. Additionally, it can bind both lipid-soluble and water-soluble substances. Therefore, BSA has essential physiological and clinical significance [20]. BSA has been proved to interact with bioactive small molecules to form nanoparticles, reducing degradation, improving solubility and stability and increasing bioavailability of bioactive small molecules [21]. The most abundant whey protein in milk is β-Lg, which accounts for around 10% of total milk protein and 50% of total whey protein. Its nutritional and functional qualities are essential in the food industry [12,22,23]. β-Lg is a globular transporter protein consisting of 162 amino acid residues with a molecular weight of 18.3 kD. It has a hydrophobic internal cavity in its molecular structure that allows it to bind to small hydrophobic molecules [24]. According to the previous studies, small molecules binding to β-Lg have effects on the secondary structure of β-Lg [25,26]. β-Lg is an appealing bioactive compound delivery carrier that improves bioavailability [16]. The second most abundant whey protein in milk is α-La. A big α-helix domain and a small β-sheet domain are joined by a disulfide bond in the secondary structure of α-La [27]. The molecular weight of α-La is approximately 14.2 kDa, and it contains many essential amino acids and a high proportion of tryptophan, lysine, cysteine and branched-chain amino acids [28]. The tertiary structures of whey protein including BSA, β-Lg and α-La were presented in Figure 1a–c.

In recent years, several studies on the structure of BSA/β-Lg/α-La and its interactions with various food functional ingredients. In the daily diet, whey protein often coexists with a variety of food functional ingredients such as polyphenols, vitamins, polysaccharides and proteins, and it is inevitable that whey protein and functional ingredients will affect or interact with each other [29]. According to reports, proteins and phenolic compounds can combine to produce complexes with a variety of structural and physicochemical characteristics [19]. Al-Shabib et al. (2020) reported that catechins could bind to β-Lg through hydrophobic action [30]. TFDG interacted with lactoferrin via static quenching [31]. Fucoxanthin is an algal pigment and belongs to one of the lutein. Similar to theaflavin, fucoxanthin is a small molecule polyhydroxy compound with a variety of physiological activities, but has a low bioavailability similar to theaflavin. Fucoxanthin could form nano complexes with whey proteins through non-covalent interactions, and all binding processes were spontaneous [19]. These findings provide a theoretical basis for the present study, which is beneficial for us to elucidate the reaction properties between TF1 and whey protein and improve the bioavailability of TF1. However, there are no systematic studies on interaction mechanisms between whey protein (BSA/β-Lg/α-La) and TF1. 

In this study, ultraviolet-visible (UV) absorption, fluorescence, circular dichroism (CD) spectroscopy, and molecular simulation docking techniques were used to investigate the interaction mechanisms of TF1 with the three major components of whey protein (BSA/β-Lg/α-La) in an aqueous solution. With the development and gradual diversification of the dairy market, the production of whey protein as a high-quality functional protein ingredient is increasing year by year. TF1 is a natural antioxidant with a variety of health functions and is an important food additive. The study of the interaction between theaflavin and whey protein to improve its biological activity and other additional nutritional values, thus enabling it to meet the increasing requirements of the food industry for functional ingredients, is of great importance to expand its application areas and to rationalize the effective use of raw milk resources. Therefore, understanding the molecular level binding mode of whey protein with TF1 is crucial for mechanism analysis and application of black tea in dairy product development. 

## 2. Materials and Methods

### 2.1. Chemical Reagents

TF1 (≥98%) was purchased from Chengdu Biopurify Phytochemicals Co., Ltd. (Chengdu, China). BSA (≥96.00%), β-Lg (≥96.03%) and α-La (≥98.40%) standards were purchased from Sigma-Aldrich Company (St. Louis, MO, USA). Phosphate buffer solution (PBS) 0.01 M was used to prepare the BSA, β-Lg, and α-La solutions (pH 7.4). All of the other chemicals used were of analytical reagent grade. 

### 2.2. Preparation of Stock Solution

BSA, β-Lg, α-La and TF1 powder were all dissolved in PBS (0.01 M), which had a pH of 7.4. TF1 was dissolved for 10 min and stored away from light. To ensure that the protein was completely hydrated, BSA, β-Lg, and α-La solutions were mixed at room temperature for two hours (400 rpm) and then refrigerated overnight. The final concentrations of BSA, β-Lg and α-La were 5.0 μM and TF1 was 0 μM, 2.5 μM, 5.0 μM, 7.5 μM, 10.0 μM, 12.5 μM, 15.0 μM. All the experimental solutions were freshly prepared and used.

### 2.3. Fluorescence Spectroscopy Measurements

A Hitachi F-4500 fluorescence spectrometer was used to measure fluorescence. The final concentrations of BSA, β-Lg and α-La in all solutions were prepared to be 5.0 μM, while the concentrations of TF1 in the solutions ranged from 0.0 to 15.0 μM, respectively. The excitation slit was 5 nm wide, and the excitation wavelength was adjusted at 280 nm. The fluorescence emission spectra of reaction solution was measured in the range of 290–450 nm at a temperature settings of 298 K. 

The synchronous fluorescence spectra of protein in the presence of different concentrations of TF1 were recorded in the excitation wavelength range of 250–320 nm at wavelength intervals (Δλ = λ*_emission_* − λ*_excitation_*) of 15 nm and 60 nm, respectively. 

Fluorescence excitation-emission matrix (EEM) spectra were collected at excitation wavelengths of 200–500 nm (every 2 nm) and scanned at a rate of 12,000 nm/min. The scanning parameters and sample solutions were consistent with the fluorescence experiments described above.

### 2.4. Ultraviolet-Visible (UV-Vis) Absorption Spectra Analysis

UV-vis absorption spectra were analyzed using a T6PC UV-vis spectrophotometer (Persee, Beijing, China). The absorption spectra of whey proteins were measured after the addition of different concentrations of theaflavin. The final concentrations of BSA, β-Lg and α-La were 5 μM and the final concentrations of theaflavin were 0 μM, 2.5 μM, 5.0 μM, 7.5 μM, 10.0 μM, 12.5 μM and 15.0 μM. The samples were recorded at 298 K and measured in a 1.0 cm path length quartz cuvette with a scanning range of 190–450 nm. PBS at pH 7.4 was used as a blank control.

### 2.5. Circular Dichroism (CD) Analysis

CD spectra were determined using a Jasco-815 spectrophotometer (Tokyo, Japan) at 298 K with a constant nitrogen flush. Solutions of BSA, β-Lg and α-La with a final concentration of 5 μm were prepared in PBS solution (pH 7.4) [32]. The prepared protein solutions were measured in the absence and presence of 15.0 μM TF1, with a scan range of 190–250 nm, a scan rate of 60 nm/min, a sampling interval of 0.5 s and a bandwidth of 1.0 nm [19].

### 2.6. Molecular Docking Study

The molecular docking of TF1 with whey protein including BSA, β-Lg and α-La were studied by simulation using AutoDock 4.2 software. The crystal structures of whey protein including BSA, β-Lg and α-La were available in the RSCB Protein Data Bank database (https://www.rcsb.org/, accessed on 3 January 2022). The TF1 molecular model, which was downloaded from PubChem’s website and created using ChemDraw software (PerkinElmer Informatics, Inc., Waltham, MA, USA), provided as the ligands in the docking process [31]. All water molecules had to be removed, Kollman charges added, and polar hydrogen bonds added before the docking simulations could begin. The Lamarckian genetic method was used to set the search parameters, and the spatial parameters were changed to cover as many protein molecules as possible for the simulation experiment [33]. The conformations of TF1 ligands with whey protein macromolecules including BSA, β-Lg and α-La were calculated, and the conformation with the lowest binding free energy was chosen. Analysis of hydrophobic forces and interacting amino acid residues between TF1 and BSA using LigPlus ^+^.

### 2.7. Statistical Analysis

Three replicated parallel experiments were used to generate the test results (mean ± SD). The figures were obtained using Origin 2019 (Microcal, Northampton, MA, USA). The experimental data was analyzed by SPSS 21.0 software (SPSS Inc., Chicago, IL, USA) using analysis of one-way variance (ANOVA) and Duncan’s multiple range test. Statistical significance between samples was set at *p* < 0.05.

## 3. Results and Discussion

### 3.1. Fluorescence Emission Spectra

Fluorescence quenching is a commonly applied method to study the mechanism of ligand-protein interactions [34]. Both static and dynamic quenching mechanisms, which involved collisional molecule diffusion and steady-state complex formation, can cause fluorescence to quenching [35]. Fluorescence quenching occurs only when the ligand binds to the protein and affects the emission of the intrinsic fluorophore. Changes in the emission peak can be used to determine some important information about the protein structure [36].

**Figure 2:**
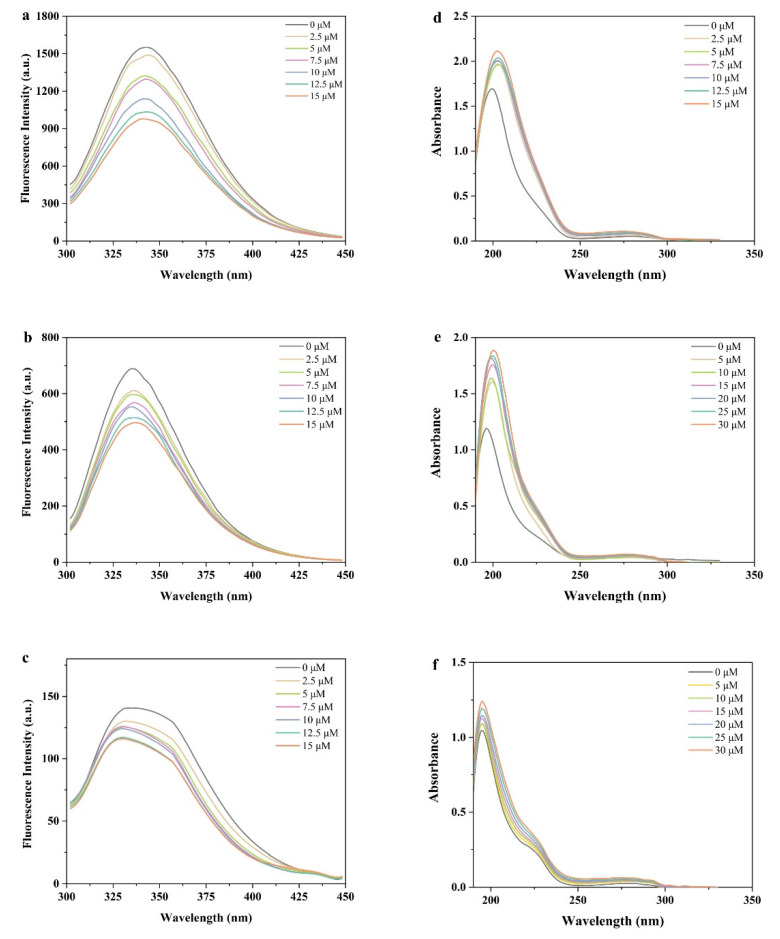
Effect of different concentrations of TF1 on fluorescence spectrum of BSA (**a**), β-Lg (**b**), α-La (**c**) and UV absorption spectra of BSA (**d**), β-Lg (**e**), α-La (**f**) at 298 K and pH 7.4.

According to Figure 2a–c, there was a concentration dependence in that BSA, β-Lg and α-La fluorescence intensity gradually decreased with the TF1 concentration increasing. Tryptophan (Trp), tyrosine (Tyr), and phenylalanine (Phe) are aromatic amino acids found in BSA that have intrinsic fluorescence properties [37]. The fluorescence emission peak of BSA at 340 nm offered information on Trp and Tyr residues at an excitation wavelength of 280 nm [38]. The results indicated a strong interaction between TF1 and BSA, and similar experimental results have been found for flavonoids (naringenin, hesperidin and apigenin) with BSA, which occurs through evidence of fluorescence quenching [39]. β-Lg contains two tryptophan residues, Trp-19 and Trp-61. Trp-19 produces 80% of the total fluorescence and is in a polar environment, whereas Trp-61 contributes less and is only partially exposed to aqueous solvents [26]. The maximum peak location of β-Lg was close to the wavelength of 340 nm, and the addition of TF1 gradually reduced the fluorescence intensity value of β-Lg. Figure 2b portrays that the highest fluorescence of β-Lg without TF1 was at 337 nm, which was consistent with earlier studies [30,40]. Tryptophan-related fluorescence, including Trp-26, Trp-60, Trp-104, and Trp-118. The intensity of α-La-TF1 complex fluorescence decreased as TF1 concentration increased. The results suggested that tryptophan residues in α-La were wrapped in TF1, which may reduce its fluorescence. The results indicated that the addition of TF1 changed the polarity of the microenvironment around Trp and Tyr residues in BSA, β-Lg and α-La. Diao et al. (2021) reported similar changes in fluorescence quenching when kaempferol formed a complex with α-La [41]. At a TF1 concentration of 15 μM, the intrinsic fluorescence intensity values of BSA, β-Lg and α-La decreased by 39.03%, 27.64% and 13.75%, respectively. The results demonstrated that TF1 could interact with BSA, β-Lg and α-La to form new complexes [42]. 

### 3.2. UV–Vis Spectroscopy Analysis

In order to better understand the molecular interactions between TF1 and whey proteins, UV spectroscopy has been widely employed to monitor electronic transitions from the ground to excited states. This study investigated the structural changes of proteins using UV absorption spectroscopy and confirmed the formation of BSA-TF1, β-lg-TF1 and α-La-TF1 complexes [43,44]. Dynamic quenching typically only changes the fluorophore’s excited state rather than its absorption spectrum. In contrast, static quenching frequently modifies the fluorophore’s absorption spectra [45]. Figure 2d–f displays the UV absorption spectra of whey protein including BSA, β-lg and α-La with 0–15 μM TF1. 

Whey protein including BSA, β-Lg and α-La all contained two absorption peaks, one peak at 210–230 nm that reflecting the structure of bones, and the other peak at 280 nm that resulted from the presence of tyrosine and tryptophan amino acid residues in the protein. Strong interactions between the protein and other molecules in mixed co-solutes can change these absorption peaks [46,47,48]. While compared to the intensity of the protein absorption peak, the TF1 absorption peaks in the protein UV spectrum were negligible. As the concentration of TF1 gradually increased, the amino residues of whey protein also showed a significant increase in UV absorption intensity at 280 nm. Moreover, with the TF1 concentration increasing, the intensity of weak peak increased as well. The UV absorption spectra of the BSA-TF1, β-Lg-TF1 and α-La-TF1 complex systems exhibited a minor blue shift, indicating that the presence of TF1 induced a slight alteration in the polarity of the BSA, β-Lg and α-La. These findings proved that binding with TF1 might alter the conformation of the whey protein including BSA, β-Lg and α-La. The changes in the intensity of the absorption supported the complex’s formation between TF1 and three whey protein [49]. The results were consistent with previous findings, and it has been widely reported that polyphenols induced bathochromic-shift for whey proteins [50,51]. 

### 3.3. Influence of TF1 Binding on the Structure of BSA/β-Lg/α-La

#### 3.3.1. Synchronous Fluorescence Spectra

Synchronous fluorescence spectroscopy is commonly used to evaluate the impact of ligands on the tertiary structure of macromolecular proteins. When the difference between excitation and emission (Δλ = λ*_emission_* − λ*_excitation_*) is fixed at 15 nm and 60 nm, respectively, synchronous fluorescence spectroscopy could provide effective characterisation of Trp residues and Tyr residues [52].

**Figure 3:**
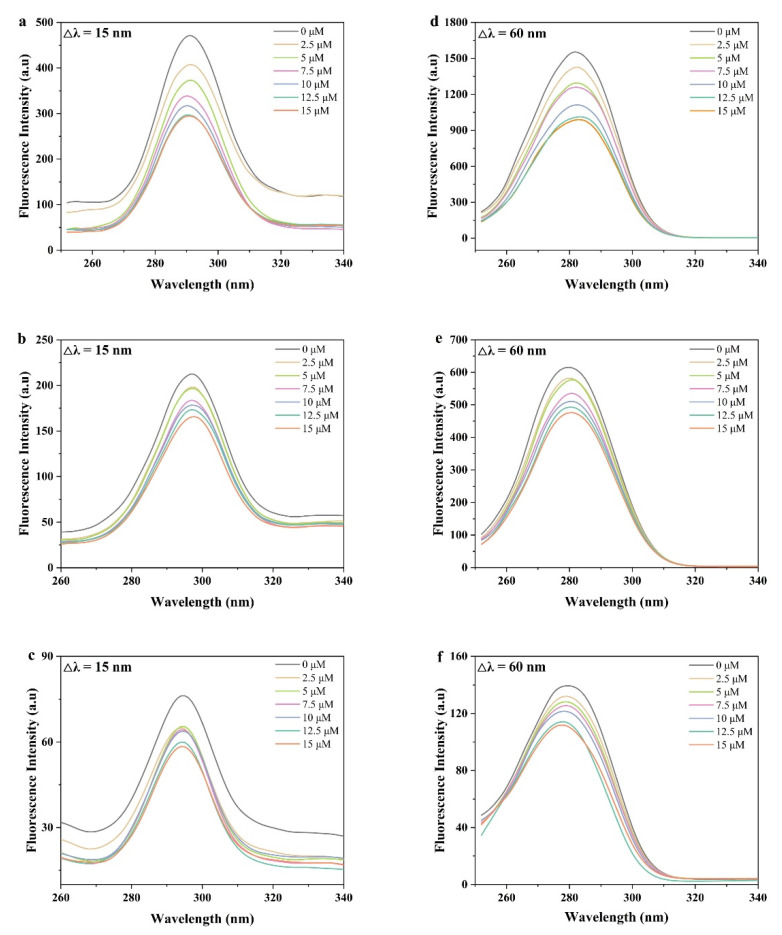
Synchronous fluorescence spectra of BSA (**a**,**d**), β-Lg (**b**,**c**) and α-La (**d**,**e**) in the presence of different concentrations of TF1 at 298 K and pH 7.4. Δλ is the interval between excitation and emission wavelength.

Figure 3 reveals that the fluorescence intensity of BSA, β-Lg and α-La decreased with the TF1 addition concentration increasing, demonstrating that the Trp residue’s (Δλ = 60 nm) and Tyr residue’s (Δλ = 15 nm) fluorescence quenching after TF1 binding to whey protein was identical. The intrinsic quenching of fluorescence was caused by Tyr and Trp residues [53]. According to the results, the Trp residue contributes more to the intrinsic fluorescence quenching than the Tyr residue (Figure 3). The fluorescence quenching intensity of the Trp residues (Δλ = 60 nm) was substantially stronger than that of the Tyr residue (Δλ = 15 nm). The structure of the Try microregion was changed, with increased hydrophobicity and decreased polarity surrounding the residues, which is compatible with the fluorescence emission spectra analyzed above. In summary, it was concluded that TF1 interacted with BSA, β-Lg and α-La to produce protein-TF1 complexes.

#### 3.3.2. Fluorescence Excitation-Emission Matrix (EEM) Spectra

**Figure 4:**
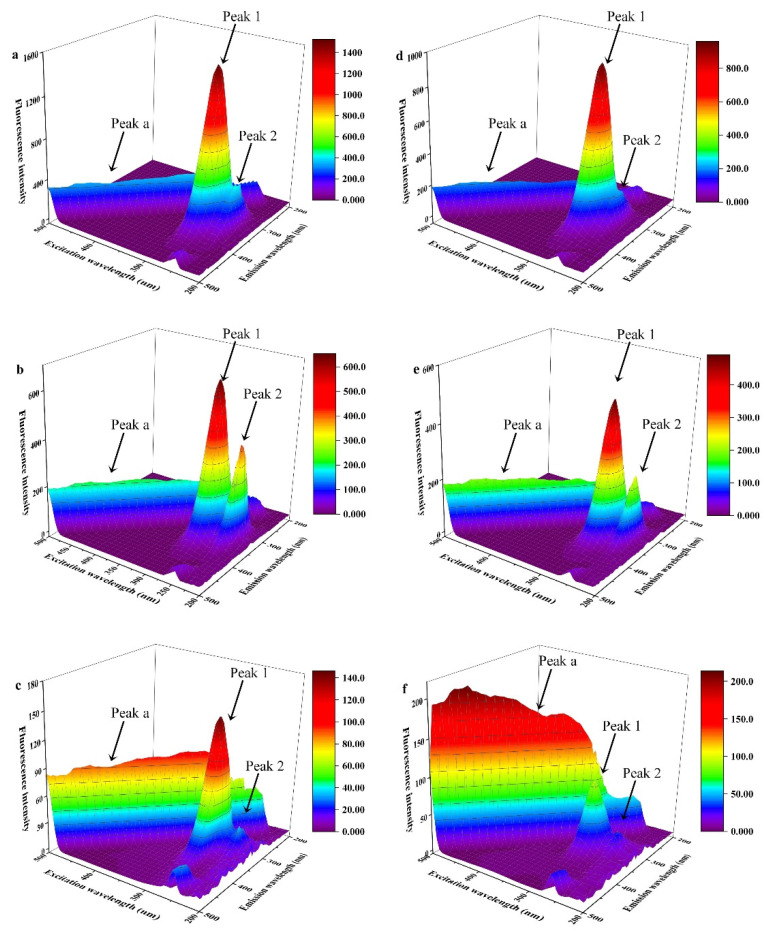
Three-dimensional fluorescence of BSA (**a**), β-Lg (**b**), α-La (**c**), BSA-TF1 (**d**), β-Lg-TF1 (**e**) and α-La-TF1 (**f**) systems at 298 K and pH 7.4. [BSA] = [β-Lg] = 5 μM, [TF1] = 15 μM.

EEM spectroscopy is a useful method for gathering precise data on changes in protein structure [54]. EEM fluorescence spectroscopy was used to examine the conformational changes that occurred in whey proteins after interacting with TF1 molecules. Figure 4 portrays that peak 1 was related to the spectral characteristics of Trp and Tyr residues, peak 2 mainly reflected the fluorescence spectral behavior of the peptide backbone structure and its intensity was related to the protein secondary structure, peak a was a Rayleigh scattering peak [53]. According to Table 1, Peak 1 in the complexes of BSA-TF1, β-Lg-TF1, and α-TF1 showed a drop in fluorescence intensity, demonstrating a strong interaction between whey protein and TF1. Further evidence that the presence of TF1 caused a significant loosening and alteration in the unfolding of the protein backbone was provided by the decrease in the fluorescence intensity of peak 2 [55]. These findings were in line with earlier findings that plant polyphenolic chemicals interact with globular proteins and cause protein unfolding [42]. 

### 3.4. Circular Dichroism (CD) Spectroscopy Analysis

**Figure 5:**
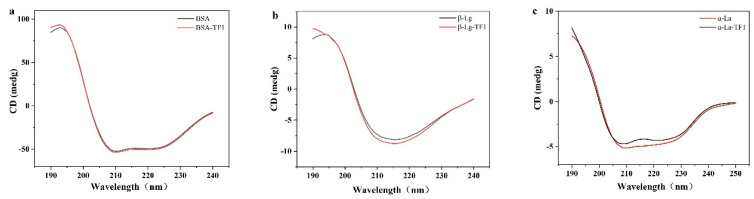
Circular dichroism (CD) spectra of free BSA (**a**), β-Lg (**b**) and α-La (**c**) and in the presence of TF1 (15 μM) at 298 K and pH 7.4.

A vital technique for analyzing the protein’s secondary structure is CD spectroscopy [56]. Changes in the secondary structure of whey protein (BSA/β-Lg/α-La) were examined using CD spectroscopy (Table 2). The CD intensity of proteins decreased slightly after the addition of TF1, demonstrating that TF1 significantly altered the protein’s structural makeup. The secondary structural components of natural BSA were 48.3% α-helix, 16.8% β-turn, 9.2% β-sheet, and 25.6% random coil. After the addition of TF1 to BSA, the α-helix content increased to 50.7%, while the β-sheet, as well as the β-turn structure content, reduced to 7.5% and 15.8%, respectively. The content of the α-helix and β-turn reduced from 39.7% to 37.8% and 25.5% to 23.2% when TF1 interacted with β-Lg, while the content of the β-sheet and random coil increased from 7.6% to 10.8% and 27.2% to 28.2%, respectively. It was evident that TF1 might alter the skeletal structure of β-Lg and induce the secondary structure to unfold due to the decrease in α-helix content and rise in β-sheet content. Li et al. (2018) reported similar findings in their investigation of the binding interaction between β-Lg and flavonoids of various structural types [42]. The α-La initially has two negative peaks at 208 nm and 222 nm. These two negative peaks are typical α-helical protein structures that are involved in the n→π*d transfer [57]. These data demonstrate that combining TF1 with β-Lg tends to make the β-sheet more ordered and compact. The secondary conformation of α-La was altered following binding to TF1 as shown in Figure 5c. The addition of TF1 increased α-helix and random coil from 38.5% to 40.7% and 33.7% to 34.3%, and decreased β-turn and β-sheet from 6.4% to 5.2% and 21.5% to 19.8%, respectively. Similar findings were made in studies of the interaction of kaempferol with α-La. Kaempferol modified the secondary structure of α-la, forming a new complex [41]. The results of the study implied that the secondary structure of the BSA, β-Lg and α-La were changed after the interaction of three whey proteins with TF1. The different changes in the secondary structure of TF1 upon complexation with BSA/β-Lg /α-La may be due to the different binding sites of TF1 to BSA, β-Lg and α-La. Similar findings were reported in previous literature [17,19,29]. The studies have revealed that the interaction of polyphenols with proteins tends to change the secondary structure of proteins, making the protein structure disordered or ordered, which may depend on the characteristics of the protein and polyphenols used to prepare the complexes [58,59]. 

### 3.5. Molecular Docking Analysis

Molecular docking technology is a computational method that simulates the binding process of a ligand at the receptor binding site [60]. Molecular docking is an effective method for identifying the mode of the binding or binding force of a ligand-protein complex. The structure of the ligand complex is simulated and the free energy of binding determines the binding strength. Binding energy is also important in considering protein-ligand interactions, with low binding energies being considered more stable [61].

**Figure 6:**
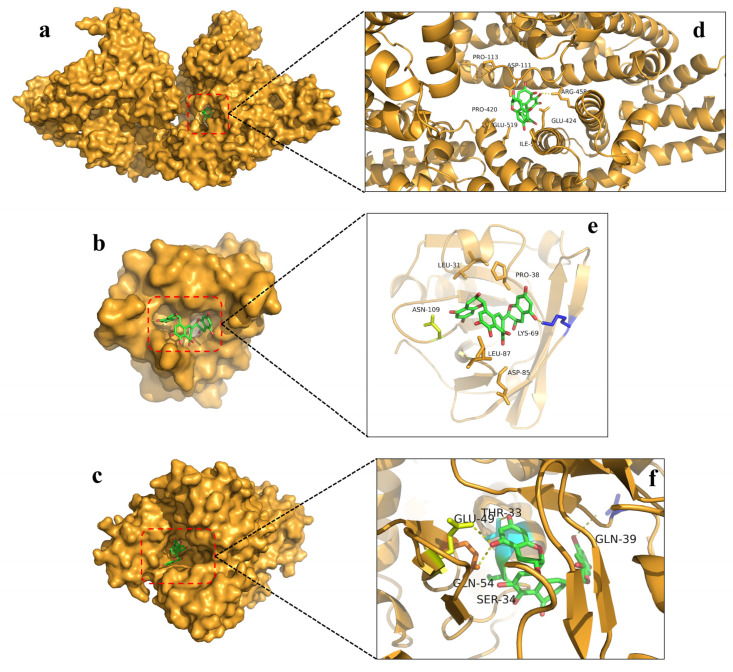
General overview (**a**–**c**) and local overview (**d**–**f**) of the best-ranked docking pose of TF1 binding with BSA (**a**,**d**), β-Lg (**b**,**e**) and α-La (**c**,**f**).

Based on the results of the experiments, the binding sites of TF1 to BSA/β-Lg/α-La were predicted using molecular docking simulations. Figure 6 depicts the results of the binding pattern simulations for molecular docking. The optimal position with the least binding energy produced the optimum build. The simulated docking model with the lowest binding energy of TF1 to BSA was illustrated in Figure 6d, in which the BSA-TF1 complex is the most stable with the lowest binding energy of −10.1 kcal/mol. Pro-420 and Arg-45 were involved in the interaction of TF1 with BSA (Figure 6c), and hydrogen bonding forces were present during the interaction. Therefore, TF1 was able to interact with BSA in a binding manner. Hydrophobic interactions and hydrogen bonding play a crucial role in the binding process of TF1 to BSA. 

**Figure 7:**
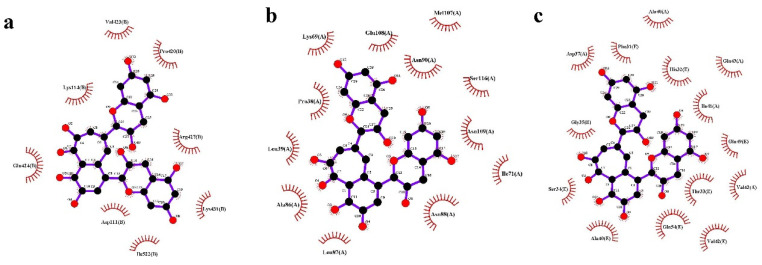
Various interactions and amino acid residues involved in stabilizing protein-TF1 complex. BSA-TF1 complex (**a**), β-lg-TF1 complex (**b**) and α-La-TF1 complex (**c**).

In addition, further analysis by LigPlus ^+^ software revealed interactions between amino acid residues such as Val-423, Pro-420, Arg-427, Lys-431, Ile-522, Asp-111, Glu-424 and Lys-114 around TF1 when bound to BSA (Figure 7a). The results indicated that both hydrogen bonding and hydrophobic forces are involved in the stabilization of BSA-TF1 complexes. The mechanism by which β-Lg transports small hydrophobic molecules is to use the hydrophobic areas of the β-Lg molecule to bind to the small hydrophobic molecules, thus forming a complex to increase the solubility of the small hydrophobic molecules. It has been demonstrated that hydrophobic small molecules bind to β-Lg at three different locations: inside the β-barrel structure (inside the Calyx structure), on the surface of the β-Lg molecule, and at the interface of the β-Lg dimer [62]. The binding energy of TF1 to β-Lg was −8.4 kcal/mol, and its optimal binding model was shown in Figure 6b. Asn-109 and Lys-69 were involved in the binding interaction of β-Lg with TF1 (Figure 6e), and hydrogen bonding forces were present during the interaction. The TF1 molecule is surrounded by amino acid residues: Met-107, Glu-108, Asn-90, Ser-116, Asn-109, Ile-71, Asn-88, Leu-87, Ala-86, Leu-39, Pro-38, Lys-69 (Figure 7b). The optimal binding mode of TF1 to α-La is shown in Figure 6c, with a binding energy of -10.4 kcal/mol. The formation of the α-La-TF1 complex involved the amino acid residues Thr-33, Gln-39, Ser-34, Gln-54, and Glu-49, with hydrophobic contacts and hydrogen bonding primarily occurring between TF1 and α-La interactions (Figure 6f). Further analysis by LigPlus ^+^ software showed the amino acid residues Ala-40, Phe-31, His-32, Ile-41, Gln-43, Glu-49, Thr-33, Val-42, Gln-54, Val-42, Ala-40, Ser-34, Gly-35 and Asp-37 around TF1 upon binding to α-La (Figure 7c). The results of the simulated docking model’s binding energy measurements are all negative, which suggests that TF1′s reaction with BSA, β-Lg, and α-La happens spontaneously. This result explained the differences in the secondary structures of the BSA-TF1, β-Lg-TF1 and α-La-TF1 complexes. TF1 has different binding sites to BSA, β-Lg and α-La, respectively. Hydrophobic contacts and hydrogen bonding were the main driving forces in the formation of the complexes, further verified the results of the fluorescence spectroscopy analysis [17,59]. 

## 4. Conclusions

In this study, the main polyphenol TF1 in black tea was investigated for its interaction mechanism with whey protein including BSA, β-Lg and α-La. The results demonstated that TF1 and whey protein (BSA/β-Lg/α-La) can assemble spontaneously. According to measurements of intrinsic fluorescence and UV-vis absorption spectra analysis, three whey protein BSA/β-Lg/α-La interacted with TF1 and quenched the fluorescence intensity through a static quenching. The secondary structure of whey protein including BSA, β-Lg and α-La altered after contact with TF1, according to the CD spectroscopy investigation. Studies using molecular docking and simulations further demonstrated that TF1 interacted with whey proteins including BSA, β-Lg and α-La. TF1 has different binding sites to BSA, β-Lg and α-La, respectively. Hydrogen bonds and hydrophobic interactions have an important influence in stabilizing the development of the whey protein-TF1 complex systems. The binding energies were −10.1 kcal mol^−1^, −8.4 kcal mol^−1^ and −10.4 kcal mol^−1^, respectively. The research in this thesis is based on the exploration of protein-polyphenol interactions. The study and understanding of the interaction between whey protein and TF1 are important for improving protein function, increasing the bioavailability of TF1 and improving the processing and manufacture of foods containing whey protein and TF1. It also provides new insights into the relationship between TF1 and whey protein and is conducive to developing and utilizing TF1 as a natural and functional additive to dairy products. This research has contributed to the creation of polyphenol-protein based carriers, laying the groundwork for the development of foods in which polyphenols and proteins co-exist, thus better exploiting the beneficial effects of polyphenols and protein ingredients. Subsequently, on the basis of the known structural and other properties of proteins and polyphenols, regularities are summarised and protein-polyphenol interactions are accurately predicted, thus establishing new strategies for the molecular processing of food products for efficient design of future foods. Additionally, future research should further focus on the effects of diverse food systems and processing methods on TF1 and whey protein interactions, as well as the physicochemical stability, functional characteristics and bioavailability of the complexes in vitro or in vivo.

## Figures and Tables

**Table 1:** Three-dimensional fluorescence spectral characteristic parameters of BSA, β-Lg, and α-La their TF1 complexes.

Compound	Peak 1 (λex/λem)	Intensity	Peak 2 (λex/λem)	Intensity
BSA	280.0/340.0	1521.0	235.0/340.0	447.4
BSA-TF1	280.0/340.0	958.8	235.0/340.0	215.8
β-Lg	280.0/335.0	653.0	235.0/335.0	410.0
β-Lg-TF1	280.0/335.0	491.5	235.0/330.0	239.3
α-La	280.0/330.0	147.3	230.0/335.0	36.7
α-La-TF1	285.0/330.0	91.76	230.0/335.0	23.6

[BSA] = [β-Lg] = [α-La] = 5 μM, [TF1] = 15 μM.

**Table 2:** CD spectra parameters of BSA/β-Lg/α-La in the absence and presence of TF1.

Sample	α-Helix (%)	β-Sheet (%)	β-Turn (%)	Random Coil (%)
BSA	48.3 ± 0.2	9.2 ± 0.0	16.8 ± 0.2	25.6 ± 0.1
BSA-TF1	50.7 ± 0.4	7.5 ± 0.0	15.8 ± 0.2	26.0 ± 0.2
β-Lg	39.7 ± 0.1	7.6 ± 0.1	25.5 ± 0.2	27.2 ± 0.0
β-Lg-TF1	37.8 ± 0.2	10.8 ± 0.1	23.2 ± 0.1	28.2 ± 0.2
α-La	38.5 ± 0.1	6.4 ± 0.1	21.5 ± 0.1	33.7 ± 0.1
α-La-TF1	40.7 ± 0.2	5.2 ± 0.0	19.8± 0.1	34.3 ± 0.2

Results are expressed as mean ± standard deviation (*n* = 3).

## Data Availability

Not applicable.

## References

[1] Xu J., Wei Y., Li F., Weng X., Wei X. (2022). Regulation of Fungal Community and the Quality Formation and Safety Control of Pu-Erh Tea. Compr. Rev. Food Sci. Food Saf..

[2] Saikia D., Boruah P.K., Sarma U. (2015). A Sensor Network to Monitor Process Parameters of Fermentation and Drying in Black Tea Production. Mapan.

[3] He H.F. (2017). Research Progress on Theaflavins: Efficacy, Formation, and Preparation. Nutr. Res..

[4] Wu Y.H., Kuraji R., Taya Y., Ito H., Numabe Y. (2018). Effects of Theaflavins on Tissue Inflammation and Bone Resorption on Experimental Periodontitis in Rats. J. Periodontal Res..

[5] Xu J., Wei Y., Huang Y., Wei X. (2023). Regulatory Effects and Molecular Mechanisms of Tea and Its Active Compounds on Nonalcoholic Fatty Liver Disease. J. Agric. Food Chem..

[6] Takemoto M., Takemoto H., Sakurada A. (2014). Synthesis of Theaflavins with *Camellia sinensis* Cell Culture and Inhibition of Increase in Blood Sugar Values in High-Fat Diet Mice Subjected to Sucrose or Glucose Loading. Tetrahedron Lett..

[7] Tanaka Y., Kirita M., Miyata S., Abe Y., Tagashira M., Kanda T., Maeda-Yamamoto M. (2013). O-Methylated Theaflavins Suppress the Intracellular Accumulation of Triglycerides from Terminally Differentiated Human Visceral Adipocytes. J. Agric. Food Chem..

[8] Braud L., Battault S., Meyer G., Nascimento A., Gaillard S., De Sousa G., Rahmani R., Riva C., Armand M., Reboul C. (2016). Antioxidant Molecules of Tea (*Camellia sinensis*) Decrease Hepatic Lipogenesis and Steatosis in a High Fat-Sucrose Diet NAFLD Rat Model. Arch. Cardiovasc. Dis. Suppl..

[9] Fatima M., Kesharwani R.K., Misra K., Rizvi S.I. (2013). Protective Effect of Theaflavin on Erythrocytes Subjected to In Vitro Oxidative Stress. Biochem. Res. Int..

[10] Tan Q., Peng L., Huang Y., Huang W., Bai W., Shi L., Li X., Chen T. (2019). Structure–Activity Relationship Analysis on Antioxidant and Anticancer Actions of Theaflavins on Human Colon Cancer Cells. J. Agric. Food Chem..

[11] Takino Y., Imagawa H. (1964). Studies on the Mechanism of the Oxidation of Tea Leaf Catechins: Part III. Formation of a Reddish Orange Pigment and Its Spectral Relationship to Some Benzotropolone Derivatives. Biosci. Biotechnol. Biochem..

[12] Xu J., Wei Y., Huang Y., Weng X., Wei X. (2022). Current Understanding and Future Perspectives on the Extraction, Structures, and Regulation of Muscle Function of Tea Pigments. Crit. Rev. Food Sci. Nutr..

[13] Lun Su Y., Leung L.K., Huang Y., Chen Z.-Y. (2003). Stability of Tea Theaflavins and Catechins. Food Chem..

[14] Qu F., Ai Z., Liu S., Zhang H., Chen Y., Wang Y., Ni D. (2021). Study on Mechanism of Low Bioavailability of Black Tea Theaflavins by Using Caco-2 Cell Monolayer. Drug Deliv..

[15] Ali M., Keppler J.K., Coenye T., Schwarz K. (2018). Covalent Whey Protein–Rosmarinic Acid Interactions: A Comparison of Alkaline and Enzymatic Modifications on Physicochemical, Antioxidative, and Antibacterial Properties. J. Food Sci..

[16] Tavares G.M., Croguennec T., Carvalho A.F., Bouhallab S. (2014). Milk Proteins as Encapsulation Devices and Delivery Vehicles: Applications and Trends. Trends Food Sci. Technol..

[17] Gong S., Yang C., Zhang J., Yu Y., Gu X., Li W., Wang Z. (2021). Study on the Interaction Mechanism of Purple Potato Anthocyanins with Casein and Whey Protein. Food Hydrocoll..

[18] Oancea A.-M., Hasan M., Vasile A.M., Barbu V., Enachi E., Bahrim G., Râpeanu G., Silvi S., Stănciuc N. (2018). Functional Evaluation of Microencapsulated Anthocyanins from Sour Cherries Skins Extract in Whey Proteins Isolate. LWT-Food Sci Technol..

[19] Zhu J., Sun X., Wang S., Xu Y., Wang D. (2017). Formation of Nanocomplexes Comprising Whey Proteins and Fucoxanthin: Characterization, Spectroscopic Analysis, and Molecular Docking. Food Hydrocoll..

[20] Sułkowska A. (2002). Interaction of Drugs with Bovine and Human Serum Albumin. J. Mol. Struct..

[21] Mishra V., Mahor S., Rawat A., Gupta P.N., Dubey P., Khatri K., Vyas S.P. (2006). Targeted Brain Delivery of AZT via Transferrin Anchored Pegylated Albumin Nanoparticles. J. Drug Target..

[22] Huang Y., Wei Y., Xu J., Wei X. (2022). A Comprehensive Review on the Prevention and Regulation of Alzheimer’s Disease by Tea and Its Active Ingredients. Crit. Rev. Food Sci. Nutr..

[23] Xie H., Luo Z.C., Xi-Can L.I. (2018). Chemical Mechanism of Antioxidation of Theaflavin. Food Mach..

[24] Patel B.K., Sepay N., Mahapatra A. (2019). Curious Results in the Prospective Binding Interactions of the Food Additive Tartrazine with β-Lactoglobulin. Langmuir.

[25] Divsalar A., Saboury A.A., Mansoori-Torshizi H., Moghaddam M.I., Ahmad F., Hakimelahi G.H. (2009). Comparative Studies on the Interaction Between Bovine β-Lacto-Globulin Type A and B and a New Designed Pd (II) Complex with Anti-Tumor Activity at Different Temperatures. J. Biomol. Struct. Dyn..

[26] Kanakis C.D., Hasni I., Bourassa P., Tarantilis P.A., Polissiou M.G., Tajmir-Riahi H.-A. (2011). Milk β-Lactoglobulin Complexes with Tea Polyphenols. Food Chem..

[27] Kamau S.M., Cheison S.C., Chen W., Liu X.-M., Lu R.-R. (2010). Alpha-Lactalbumin: Its Production Technologies and Bioactive Peptides. Compr. Rev. Food Sci. Food Saf..

[28] Permyakov E.A., Berliner L.J. (2000). α-Lactalbumin: Structure and Function. FEBS Lett..

[29] Kong F., Kang S., Zhang J., Jiang L., Liu Y., Yang M., Cao X., Zheng Y., Shao J., Yue X. (2022). The Non-Covalent Interactions between Whey Protein and Various Food Functional Ingredients. Food Chem..

[30] Al-Shabib N.A., Khan J.M., Malik A., Tabish Rehman M., AlAjmi M.F., Husain F.M., Hisamuddin M., Altwaijry N. (2020). Molecular Interaction of Tea Catechin with Bovine β-Lactoglobulin: A Spectroscopic and in Silico Studies. Saudi Pharm. J..

[31] Wang M., Xu J., Han T., Tang L. (2021). Effects of Theaflavins on the Structure and Function of Bovine Lactoferrin. Food Chem..

[32] He Z., Xu M., Zeng M., Qin F., Chen J. (2016). Interactions of Milk α- and β-Casein with Malvidin-3-O-Glucoside and Their Effects on the Stability of Grape Skin Anthocyanin Extracts. Food Chem..

[33] Xu J., Wang M., Zheng Y., Tang L. (2019). Spectroscopic Technique-Based Comparative Investigation on the Interaction of Theaflavins with Native and Glycated Human Serum Albumin. Molecules.

[34] Alanazi M.M., Almehizia A.A., Bakheit A.H., Alsaif N.A., Alkahtani H.M., Wani T.A. (2019). Mechanistic Interaction Study of 5,6-Dichloro-2-[2-(Pyridin-2-Yl)Ethyl]Isoindoline-1,3-Dione with Bovine Serum Albumin by Spectroscopic and Molecular Docking Approaches. Saudi Pharm. J..

[35] Ouyang Y., Chen L., Qian L., Lin X., Fan X., Teng H., Cao H. (2020). Fabrication of Caseins Nanoparticles to Improve the Stability of Cyanidin 3-O-Glucoside. Food Chem..

[36] Zhan F., Ding S., Xie W., Zhu X., Hu J., Gao J., Li B., Chen Y. (2020). Towards Understanding the Interaction of β-Lactoglobulin with Capsaicin: Multi-Spectroscopic, Thermodynamic, Molecular Docking and Molecular Dynamics Simulation Approaches. Food Hydrocoll..

[37] Yu X., Cai X., Luo L., Wang J., Ma M., Wang M., Zeng L. (2020). Influence of Tea Polyphenol and Bovine Serum Albumin on Tea Cream Formation by Multiple Spectroscopy Methods and Molecular Docking. Food Chem..

[38] Zhang H., Deng H., Wang Y. (2020). Comprehensive Investigations about the Binding Interaction of Acesulfame with Human Serum Albumin. Spectrochim. Acta Part A Mol. Biomol. Spectrosc..

[39] Bi S., Yan L., Pang B., Wang Y. (2012). Investigation of Three Flavonoids Binding to Bovine Serum Albumin Using Molecular Fluorescence Technique. J. Lumin..

[40] Jia J., Gao X., Hao M., Tang L. (2017). Comparison of Binding Interaction between β-Lactoglobulin and Three Common Polyphenols Using Multi-Spectroscopy and Modeling Methods. Food Chem..

[41] Diao M., Liang Y., Zhao J., Zhao C., Zhang J., Zhang T. (2021). Enhanced Cytotoxicity and Antioxidant Capacity of Kaempferol Complexed with α-Lactalbumin. Food Chem. Toxicol..

[42] Li T., Hu P., Dai T., Li P., Ye X., Chen J., Liu C. (2018). Comparing the Binding Interaction between β-Lactoglobulin and Flavonoids with Different Structure by Multi-Spectroscopy Analysis and Molecular Docking. Spectrochim. Acta Part A Mol. Biomol. Spectrosc..

[43] Condict L., Kaur J., Hung A., Ashton J., Kasapis S. (2019). Combined Spectroscopic, Molecular Docking and Quantum Mechanics Study of β-Casein and Ferulic Acid Interactions Following UHT-like Treatment. Food Hydrocoll..

[44] Shpigelman A., Shoham Y., Israeli-Lev G., Livney Y.D. (2014). β-Lactoglobulin–Naringenin Complexes: Nano-Vehicles for the Delivery of a Hydrophobic Nutraceutical. Food Hydrocoll..

[45] Li X.R., Wang S. (2015). Binding of Glutathione and Melatonin to Human Serum Albumin: A Comparative Study. Colloids Surf. B Biointerfaces.

[46] Guo X., Li X., Jiang Y., Yi L., Wu Q., Chang H., Diao X., Sun Y., Pan X., Zhou N. (2014). A Spectroscopic Study on the Interaction between P-Nitrophenol and Bovine Serum Albumin. J. Lumin..

[47] Kaur J., Katopo L., Hung A., Ashton J., Kasapis S. (2018). Combined Spectroscopic, Molecular Docking and Quantum Mechanics Study of β-Casein and p-Coumaric Acid Interactions Following Thermal Treatment. Food Chem..

[48] Wu X., Lu Y., Xu H., Lin D., He Z., Wu H., Liu L., Wang Z. (2018). Reducing the Allergenic Capacity of β-Lactoglobulin by Covalent Conjugation with Dietary Polyphenols. Food Chem..

[49] Zhang Y., Zhong Q. (2013). Probing the Binding between Norbixin and Dairy Proteins by Spectroscopy Methods. Food Chem..

[50] Diao M., Liang Y., Zhao J., Zhang J., Zhang T. (2022). Complexation of Ellagic Acid with α-Lactalbumin and Its Antioxidant Property. Food Chem..

[51] Lu Y., Zhao R., Wang C., Zhang X., Wang C. (2022). Deciphering the Non-Covalent Binding Patterns of Three Whey Proteins with Rosmarinic Acid by Multi-Spectroscopic, Molecular Docking and Molecular Dynamics Simulation Approaches. Food Hydrocoll..

[52] Bobone S., van de Weert M., Stella L. (2014). A Reassessment of Synchronous Fluorescence in the Separation of Trp and Tyr Contributions in Protein Emission and in the Determination of Conformational Changes. J. Mol. Struct..

[53] Bi H., Tang L., Gao X., Jia J., Lv H. (2016). Spectroscopic Analysis on the Binding Interaction between Tetracycline Hydrochloride and Bovine Proteins β-Casein, α-Lactalbumin. J. Lumin..

[54] Peng X., Zhang G., Liao Y., Gong D. (2016). Inhibitory Kinetics and Mechanism of Kaempferol on α-Glucosidase. Food Chem..

[55] Peng W., Ding F., Peng Y.-K., Sun Y. (2014). Molecular Recognition of Malachite Green by Hemoglobin and Their Specific Interactions: Insights from in Silico Docking and Molecular Spectroscopy. Mol. Biosyst..

[56] Wu D., Yan J., Wang J., Wang Q., Li H. (2015). Characterisation of Interaction between Food Colourant Allura Red AC and Human Serum Albumin: Multispectroscopic Analyses and Docking Simulations. Food Chem..

[57] Mahanta S., Paul S. (2015). Bovine α-Lactalbumin Functionalized Graphene Oxide Nano-Sheet Exhibits Enhanced Biocompatibility: A Rational Strategy for Graphene-Based Targeted Cancer Therapy. Colloids Surf. B Biointerfaces.

[58] Liu Q., Sun Y., Cheng J., Zhang X., Guo M. (2022). Changes in Conformation and Functionality of Whey Proteins Induced by the Interactions with Soy Isoflavones. LWT.

[59] Wang Y., Zhang J., Zhang L. (2022). Study on the Mechanism of Non-Covalent Interaction between Rose Anthocyanin Extracts and Whey Protein Isolate under Different PH Conditions. Food Chem..

[60] Bi W., Wang F., Han J., Liu B., Shen J., Zhang L., Okamoto Y. (2020). Influence of the Substituents on Phenyl Groups on Enantioseparation Property of Amylose Phenylcarbamates. Carbohydr. Polym..

[61] Lakshmana Senthil S., Raghu C., Arjun H.A., Anantharaman P. (2019). In Vitro and in Silico Inhibition Properties of Fucoidan against α-Amylase and α-D-Glucosidase with Relevance to Type 2 Diabetes Mellitus. Carbohydr. Polym..

[62] Harvey B.J., Bell E., Brancaleon L. (2007). A Tryptophan Rotamer Located in a Polar Environment Probes PH-Dependent Conformational Changes in Bovine β-Lactoglobulin A. J. Phys. Chem. B.

